# MicroRNA Expression and Clinical Outcome of Small Cell Lung Cancer

**DOI:** 10.1371/journal.pone.0021300

**Published:** 2011-06-22

**Authors:** Jih-Hsiang Lee, Johannes Voortman, Anne-Marie C. Dingemans, Donna M. Voeller, Trung Pham, Yisong Wang, Giuseppe Giaccone

**Affiliations:** 1 Medical Oncology Branch, National Cancer Institute, National Institutes of Health, Bethesda, Maryland, United States of America; 2 Department of Respiratory Medicine, GROW-School for Oncology and Developmental Biology, Maastricht University Medical Center, Maastricht, The Netherlands; Queen Elizabeth Hospital, Hong Kong

## Abstract

The role of microRNAs in small-cell lung carcinoma (SCLC) is largely unknown. miR-34a is known as a p53 regulated tumor suppressor microRNA in many cancer types. However, its therapeutic implication has never been studied in SCLC, a cancer type with frequent dysfunction of p53. We investigated the expression of a panel of 7 microRNAs (miR-21, miR-29b, miR-34a/b/c, miR-155, and let-7a) in 31 SCLC tumors, 14 SCLC cell lines, and 26 NSCLC cell lines. We observed significantly lower miR-21, miR-29b, and miR-34a expression in SCLC cell lines than in NSCLC cell lines. The expression of the 7 microRNAs was unrelated to SCLC patients' clinical characteristics and was neither prognostic in term of overall survival or progression-free survival nor predictive of treatment response. Overexpression or downregulation of miR-34a did not influence SCLC cell viability. The expression of these 7 microRNAs also did not predict *in vitro* sensitivity to cisplatin or etoposide in SCLC cell lines. Overexpression or downregulation of miR-34a did not influence sensitivity to cisplatin or etoposide in SCLC cell lines. In contrast to downregulation of the miR-34a target genes cMET and Axl by overexpression of miR-34a in NSCLC cell lines, the intrinsic expression of cMET and Axl was low in SCLC cell lines and was not influenced by overexpression of miR-34a. Our results suggest that the expression of the 7 selected microRNAs are not prognostic in SCLC patients, and miR-34a is unrelated to the malignant behavior of SCLC cells and is unlikely to be a therapeutic target.

## Introduction

Small cell lung cancer (SCLC) accounts for about 10–20% of lung cancer cases and is notorious for its aggressiveness and poor survival rate [1], [2]. Despite moderate progress achieved in the past two decades, the survival rate of SCLC patients is still very poor [2], [3]. Partially due to the lack of adequate molecular biomarkers to guide treatment of SCLC, the clinical results of molecular targeted therapies such as imatinib, gefitinib, bcl-2 inhibitors, and mTOR inhibitors in the treatment of SCLC patients are disappointing [4].

MicroRNA expression correlates with biological characteristics of cancer, such as cell differentiation, aggressiveness, invasion, angiogenesis, and metastatic behavior [5], [6], [7]. For example, the miR-34 family members, miR-34a, miR-34b, and miR-34c, are direct transcriptional targets of p53 and their expression induces cell cycle arrest in cancer cell lines [8]. miR-29b acts as a tumor suppressor microRNA through restoration of a normal DNA methylation pattern [9]. Clinically, microRNA profiling has been shown to aid in the diagnosis of cancer as well as prediction of prognosis and treatment response [6], [10]. The roles of microRNAs in cancer biology and prognosis prediction in non-small cell lung cancer (NSCLC) have been widely studied. Increased miR-34a was associated with fewer relapses in a small retrospective study of resected NSCLC patients [11]. Overexpression of let-7a, possibly through suppression of the RAS oncogene [12], was shown to be related to increased overall survival in NSCLC patients [13], and was also among the protective prognostic factors preventing recurrence in surgically resected NSCLC [14]. Overexpression of “oncomirs” miR-21 and miR-155 was shown to be related to decreased overall survival in NSCLC patients [13], [15]. There is only scant data on the role of microRNA expression in SCLC, and the function of several well-documented cancer-related microRNAs in other cancer types has never been addressed in SCLC. For instance, although SCLC is characterized by frequent p53 dysfunction [16], the role of miR-34a, a p53 regulated tumor suppressor microRNA [8], [17], has never been studied in SCLC.

We recently demonstrated, by using a real-time polymerase chain reaction (PCR) -based plateform, that expression profiles of a panel of seven cancer-associated microRNAs (miR-21, miR-29b, miR-34a/b/c, miR-155, and let-7a) are neither predictive nor prognostic in NSCLC patients receiving platinum-based adjuvant chemotherapy [18]. However, in resected pancreatic cancer using the same panel of microRNAs, we showed that low expression of miR-21 was associated with increased survival following adjuvant treatment in two independent cohorts of pancreatic ductal adenocarcinoma patients [19], suggesting that expression of microRNAs and their prognostic and predictive implications are likely to be tumor specific. In the current study, we explored the role of microRNAs for the prediction of both prognosis and treatment outcome in SCLC using patient samples and SCLC cell lines. We further studied how expression of miR-34a affects the malignant behavior of SCLC cells.

## Results

### microRNA expression in SCLC tumors and lung cancer cell lines

Table 1 summarizes the main patients' characteristics, and Table S1 depicts microRNA expression in relation to clinical variables. Trends were observed in favor of higher let-7a expression in women and in younger patients (p = 0.07 and 0.13 respectively, by Wilcoxon Rank-Sum test). No significant correlations were observed between expression of microRNAs and clinical characteristics. We compared expression of the 7 microRNAs between SCLC cell lines and NSCLC cell lines. Lower expression of miR-21, miR-29b, and miR-34a was found in SCLC cell lines than in NSCLC cell lines (p<0.001 for all three microRNAs, Figure S1), which is in agreement with a previous report [20]. We further performed *in situ* hybridization of miR-34b in a SCLC tumor specimen and observed cytoplasmic expression of miR-34b and nuclear expression of nucleolar RNA U6 as expected (Figure S2).

**Table 1:** Patient characteristics and survival.

Characteristics		No.	OS	p-value
Total no. of patients		31		
Age, median(yr) (range)		63 (38–78)		
	≤ median	16	49.1	0.687
	> median	15	48.1	
Gender	male	25	52.0	0.253
	female	6	43.1	
Disease extent	Limited	18	52.7	0.007
	Extensive	13	40.4	
Chemotherapy	CEE	20	51.5	0.132
	CEV/PE	11	40.4	
Response	Complete response	8	66.4	<0.001
	Non-complete response	23	43.1	
Radiotherapy	Yes	13	58.3	0.052
	No	18	36.6	

OS: median overall survival (weeks). CEE, cyclophosphamide-epirubicin-etoposide. CEV/PE, cyclophosphamide-epirubicin-vincristine alternated with carboplatin-etoposide.

### microRNA expression is not correlated with survival of SCLC patients

The median overall survival and progression-free survival of this study cohort were 49.1 and 35.7 weeks, respectively. As expected, longer overall survival was observed in SCLC patients who had limited disease and patients who achieved complete response after treatment (Table 1 and Table S2). Among the 7 microRNAs studied, there was no survival difference between patients with high and low microRNA expression, defined by the median expression of each microRNA in tumor specimens (Table 2). This was true in both limited and extensive disease patients (Table S3).

**Table 2:** microRNA expression and survival analysis in SCLC patients.

		OS	p-value	PFS	p-value
mir21	high	51.5	0.67	38.4	0.75
	low	46.9		33.9	
mir29b	high	49.1	029	35.7	0.74
	low	46.4		32.7	
mir34a	high	49.1	0.52	34.9	0.52
	low	46.9		36.1	
mir34b	high	52.0	0.50	34.9	0.35
	low	46.4		36.1	
mir34c	high	52.7	0.71	42.0	0.67
	low	40.4		33.9	
mir155	high	51.6	0.74	35.7	0.77
	low	46.9		34.9	
let-7a	high	45.0	0.70	30.1	0.95
	low	53.1		36.1	

Cut off values were the median of each microRNA expression OS: median overall survival (weeks), PFS: median progression-free survival (weeks).

### p53 protein expression is unrelated to miR-34a/b/c expression in SCLC tumors

As miR-34a, miR-34b, and miR-34c are direct transcriptional targets of p53 [8] and bcl-2 is a target of miR-34a[21], we explored whether p53 expression affects expression of miR-34a/b/c and whether bcl-2 expression is related to miR-34a. Using specimens from the same study cohort, Dingemans *et al* demonstrated that protein expression of p53 is unrelated to survival of SCLC patients and is unrelated to protein expression of bcl-2 as well [22]. We observed that protein expression of p53 in SCLC tumors was unrelated to expression of miR-34a, miR-34b, and miR-34c (Figure S3A, B, and C). Additionally, expression of miR-34a was unrelated to expression of bcl-2 in SCLC tumors (Figure S3D).

### miR-34a expression is not related to viability of SCLC *in vitro*

**Figure 1:**
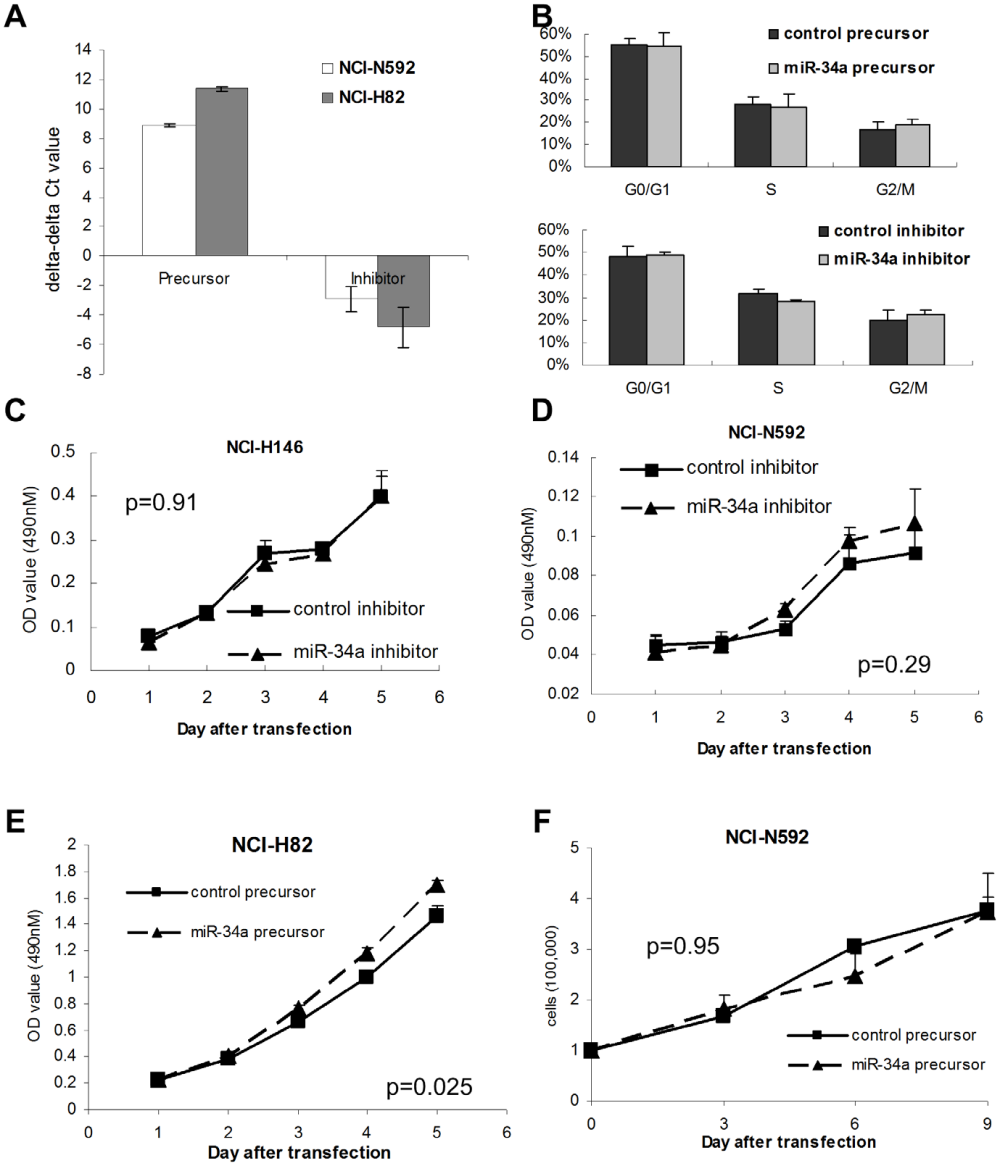
Ectopic overexpression or downregulation of miR34a in SCLC cell lines by microRNA precursor or inhibitor. (A) miR-34a expression in NCI-N592 cells and NCI-H82 cells transfected with miR-34a precursor or inhibitor. The delta-delta Ct value was calibrated to the delta Ct value of miR34a in cells transfected with control miR precursor or inhibitor, respectively. (B) Cell cycle distribution of NCI-H82 cell transfected with control miR precursor, miR-34a precursor, control miR inhibitor, and miR-34a inhibitor. (C and D) Cell growth assay of NCI-H146 (C) and NCI-N592 (D) cells transfected with miR-34a inhibitor or control miR inhibitor. (E) Cell growth assay of NCI-H82 cells transfected with miR-34a precursor or control miR precursor. (F) Cell viability of NCI-N592 cells after repetitive transfection of control miR precursor or miR-34a precursor. Cells were counted and transfected again every three days. Error bars indicate standard deviation. p-values were calculated according to cell viability 5 days after transfection with the exception of microRNA precursor in NCI-N592 cell, which was calculated according to the number of cells counted 9 days after the first transfection.

To explore the role of microRNA expression in the malignant behavior of SCLC, we focused on miR-34a because miR-34a was reported to be a prognostic survival marker [11] and to be one of the few candidates for microRNA replacement therapy in NSCLC [23], [24]. We over-expressed or down-regulated miR-34a in SCLC cells. Using a Cy3-labeled miR precursor or inhibitor to evaluate the transfection efficacy, we found that 24 hours after transfection, the transfection efficacy of miR-precursor or inhibitor in NCI-H82 SCLC cells was nearly 100% (Figure S4). High transfection efficacy was also observed in NCI-H146, NCI-N592, GLC4, A549, and NCI-H1299 cells (data not shown). We observed higher mature miR-34a expression in cells transfected with miR-34a precursor and lower mature miR-34a expression in cells transfected with miR-34a inhibitor as compared to cells transfected with control precursor and inhibitor, respectively (Figure 1A). Whereas overexpression of miR-34a induces G1 arrest in several cancer cell lines[8], G1 arrest was not observed in NCI-H82 SCLC cell transfected with miR-34a precursor (Figure 1B). Downregulation of miR-34a in NCI-H146 cells, which expressed higher level of miR-34a in comparison with the median expression level of the 14 SCLC cell lines (Table S4), and in NCI-N592 cells, which expressed lower level of miR-34a, did not influence viability of SCLC cells (Figure 1C and D). In contrast to the generally accepted tumor suppressor effect of miR-34a, overexpression of miR-34a in NCI-82 cells failed to attenuate cell growth (Figure 1E), whereas attenuation of cell growth was observed in NCI-H1299 NSCLC cells (Figure S5), as demonstrated in a previous report [23]. Unlike Wiggins *et al.* who demonstrated that repetitive transfection and long-term incubation of miR-34a decreases NCI-H226 NSCLC cancer cell viability [23], we failed to observe the effect of repetitive transfection of miR-34a on decreasing the cell viability in NCI-N592 cells (Figure 1F).

### microRNA expression does not influence drug sensitivity of SCLC cells

We first tested the correlation between microRNA expression and sensitivity of the 14 SCLC cell lines to cisplatin and etoposide. Not surprisingly, the sensitivity of SCLC cells to etoposide correlates with the sensitivity to cisplatin (correlation coefficient = 0.74, p = 0.004). We observed that the expression of the 7 microRNAs was not associated with chemosensitivity of SCLC cells to either agent (Table 3).

**Table 3:** microRNA expression and chemosensitivity to etoposide or cisplatin in SCLC cell lines.

	Etoposide	Cisplatin
	CC	p-value	CC	p-value
miR-21	0.17	0.56	0.05	0.86
miR-29b	0.41	0.14	0.19	0.51
miR-34a	0.09	0.76	−0.06	0.84
miR-34b	0.15	0.62	0.00	0.99
miR-34c	0.12	0.67	−0.09	0.77
miR-155	0.13	0.66	−0.33	0.25
let-7a	0.45	0.10	0.41	0.14

CC: correlation coefficient by Spearman method.

**Figure 2:**
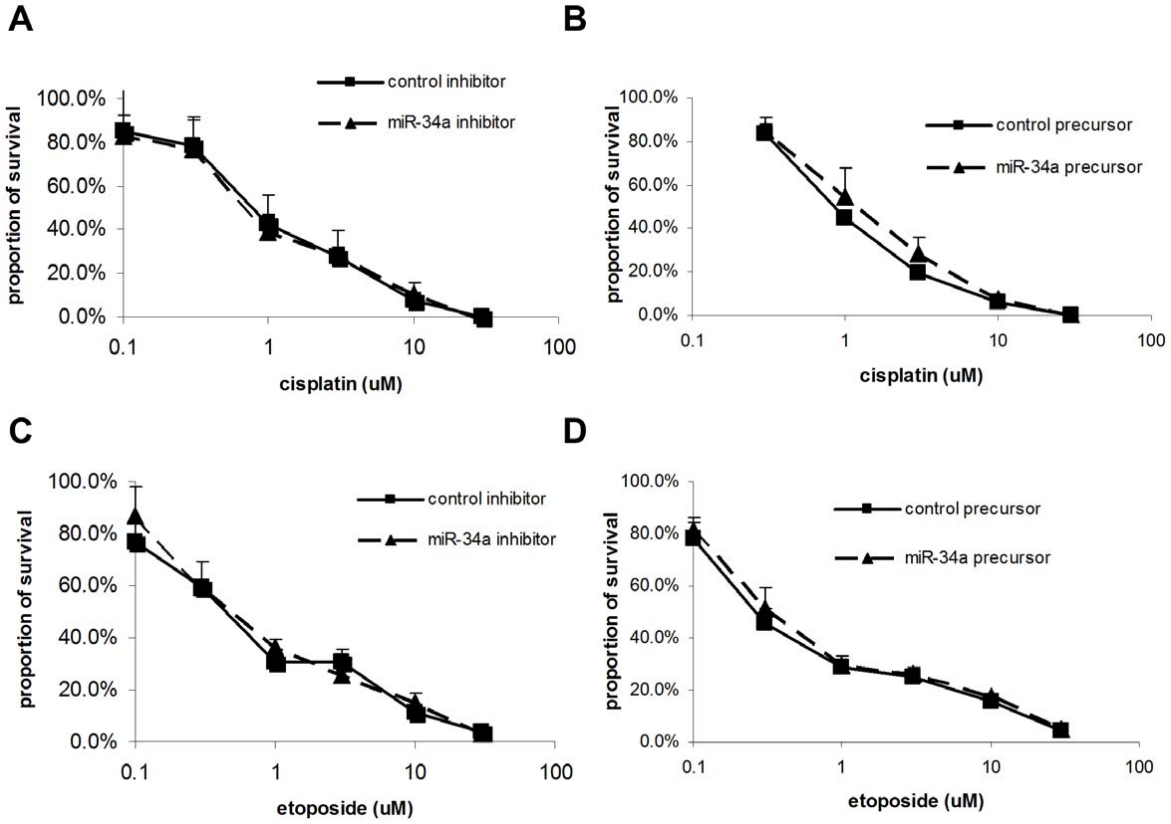
Growth inhibition assay of NCI-H82 cells treated with (A and B) cisplatin and (C and D) etoposide. Cells were transfected with the indicated oligonucleotides. Error bars indicate standard deviation of assays in triplicate.

We next evaluated whether overexpression or downregulation of miR-34a in SCLC cells would influence chemotherapy sensitivity, as miR-34a expression was reported to be related to chemotherapy resistance in a prostate cancer cell line [25]. NCI-H82 cells, which carry a silent TP53 mutation (Table S4) were transfected with control miR precursor, miR-34a precursor, control miR inhibitor and miR-34a inhibitor, and subsequently treated with cisplatin or etoposide. IC_50_ values upon cisplatin treatment were 0.9±0.02 µM, 1.19±0.3 µM, 0.83±0.15 µM, and 0.79±0.13 µM, respectively (p = 0.1 by one-way ANOVA) (Figure 2A and B). The IC_50_ values upon etoposide treatment were 0.28±0.05 µM, 0.31±0.04 µM, 0.54±0.40 µM, and 0.58±0.07 µM, respectively (p = 0.24) (Figure 2C and D). For GLC4 cells, which carry K132E mutation in the TP53 gene (Table S4), the IC_50_ values upon cisplatin treatment were 3.03±0.87 µM, 2.56±0.85 µM, 1.87±0.30 µM, and 1.92±0.61 µM, respectively (p = 0.14) (Figure S6A); the IC_50_ values upon etoposide treatment were 0.19±0.03 µM, 0.28±0.01 µM, 0.20±0.03 µM, and 0.19±0.06 µM, respectively (p = 0.08) (Figure S6B). In summary, transient overexpression or downregulation of miR-34a in SCLC cells did not affect the sensitivity to cisplatin or etoposide, regardless of the TP53 status.

### Expression of miR-34a target genes differ among cancer cell lines

**Figure 3:**
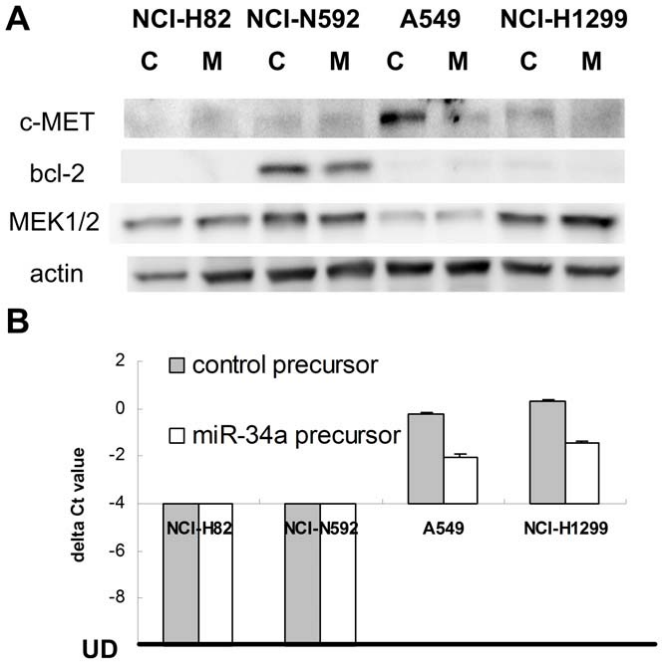
Expression of miR-34a target genes in SCLC and NSCLC cancer cell lines. (A) protein expression of miR-34a targets in cancer cell lines tranfected with control precursor (C) or miR-34a precursor (M). (B) mRNA expression of the AXL gene in cancer cells transfected with control precursor or miR-34a precursor. RNA was collected 24 hr after transfection. The expression of the gene in NCI-H82 and NCI-N592 cells were undetectable (UD).

We evaluated the expression of selected miR-34a target-genes in SCLC and NSCLC cell lines. Genes selected were MET[8], MAP2K1[26], BCL2[21], and AXL[27]. We observed an inverse correlation between miR-34a and c-MET but not bcl-2 expression in 5 SCLC cell lines (Figure S7A and B). Despite relatively lower miR-34a expression in SCLC cell lines (Figure S1), we did not observe comparatively higher c-MET expression in SCLC cell lines than in NSCLC cell lines (Figure S7A). We observed however a decreased protein expression of cMET in A549 and NCI-H1299 NSCLC cell lines transfected with miR-34a precursor (Figure 3A). Since cMET expression in NCI-H82 was undetectable, ectopic expression of miR-34a or control precursors had no effect on cMET expression, as expected. A decrease of bcl-2 protein expression was observed in NCI-N592 cells transfected with miR-34a precursor (Figure 3A). As only the MAP2K1 but not the MAP2K2 gene is the target of miR-34a, we did not detect reduced protein expression of MEK1/2 in cells transfected with miR-34a precursor; this is presumably due to redundant roles of MAP2K1 and 2. Transfection of miR-34a precursor downregulated mRNA expression of the AXL gene in A549 and NCI-H1299 NSCLC cells by more than 70% (change of delta Ct by more than 1.7), whereas the mRNA expression of the AXL gene in NCI-H82 and NCI-N592 SCLC cells was too low to be detected by real-time PCR assay (Figure 3B). In summary, we demonstrated that ectopic expression of miR-34a could down-regulate miR-34a target-genes in cells expressing the targets.

## Discussion

Here we showed the expression of 7 known cancer-associated microRNAs in SCLC tumors and cell lines as well as in NSCLC cell lines. We observed that the expression of these microRNAs was unrelated to clinical characteristics and outcome of SCLC patients. Expression of these microRNAs was unrelated to the sensitivity of SCLC cells to cisplatin or etoposide. Moreover, overexpression or downregulation of miR-34a did not influence SCLC cell viability or sensitivity to cisplatin and etoposide. We further observed that the expression of miR-34a target genes may differ among SCLC and NSCLC cell lines.

Although microRNAs have been widely studied in several cancer types, there are only a few publications addressing the role of microRNA in the malignant behavior of SCLC [20], [28], [29], [30]. Miko *et al.* compared microRNA expression between SCLC cell lines and tumor-free lung tissue from patients with chronic obstructive pulmonary disease [28]. Du *et al.* compared the microRNA expression pattern between 9 SCLC and 7 NSCLC cell lines, and one mesothelioma cell line [20], and Guo *et al.* compared a SCLC cell line, NCI-H69, with its doxorubicin-resistant sub-line [30]. Ranade *et al.* reported the only study using SCLC tumor specimens [29]; 16 microRNAs were studied for survival analysis in 25 patients, and miR-92a-2* was identified as a potential poor prognostic marker.

Although expression of the 7 microRNAs in our study was evaluated in many cancer types *in vitro*, *in vivo*, and in tumor specimens, they have not been evaluated before in relation to patient survival in SCLC. Although our study is retrospective and the patient cohort is small, this is by far the largest SCLC patient cohort and collection of SCLC cell lines investigating microRNA expression in this tumor type. In line with our previous report, a posthoc analysis of a large prospective randomized trial in NSCLC [18], the expression of the 7 microRNAs was not prognostic nor did they correlate to treatment outcome in SCLC patients. We cannot exclude the possibility that microRNAs other than the 7 microRNAs presented here may be important for SCLC, and therefore further studies may be warranted in SCLC to evaluate the prognostic value of microRNAs not included in this study.

miR-34a is a widely studied tumor suppressor microRNA that mediates apoptosis, cell cycle arrest, inhibition of proliferation, senescence, and inhibition of invasion and migration in various cancer types (review by Hermeking *et al*. [31]). The tumor suppressor effect of miR-34a on cancer cells, however, may be tumor type specific; whereas miR-34a down-regulates growth of IMR-90 fibroblast[8] as well as several NSCLC cell lines[23], Li *et al.* showed that overexpression of miR-34a does neither affect cell proliferation, cell cycle distribution, nor apoptosis in a hepatocellular carcinoma cell line [32], and Pang *et al.* demonstrated that overexpression of miR-34a has no effect on proliferation of cervical cancer cell lines Hela and SiHa as well as choriocarcinoma cell line JAR [33]. miR-34a is a direct transcriptional target of p53 [8], and mutations of the TP53 gene have been found in more than 75% of SCLC specimens [16]. A trend favoring lower expression of miR-34a in SCLC cell lines compared to tumor-free lung tissue has been observed previously [28]. In agreement with our finding that ectopic miR-34a expression did not down-regulate cell growth or induce G1 arrest, miR-34a expression did not correlate with disease status (Table S1) and progression (Table 2) of the SCLC patients. Intriguingly, all the SCLC cell lines tested in this study expressed low level of cMET and Axl, two miR-34a target genes. It is plausible that the growth suppression effect of miR-34a may be tissue- and disease-specific, dictated possibly by the level of intrinsic miR-34a target-gene expression in which cells with low levels of miR-34a target-gene expression, such as SCLC cells, may render miR-34a functionally impotent.

In conclusion, the expression of 7 cancer-associated microRNAs did not correlate to clinical outcomes of SCLC patients. miR-34a expression was also not related to malignant behavior of SCLC cancer cells and is thus unlikely to be a candidate therapeutic target for this disease.

## Materials and Methods

### Patients and ethics statement

Thirty-one formalin fixed, paraffin-embedded (FFPE) tumor samples from patients who were included in a phase III trial of the North-Holland Oncology group [22] were obtained from the VU University Medical Center, Amsterdam, Netherlands, upon approval of the Institutional Review Board [22]. Written informed consents were obtained from all participants.

### Cell lines

A panel of 14 SCLC and 26 NSCLC cell lines were studied and are listed in the Materials S1. All cancer cell lines were maintained in RPMI-1640, supplemented with 10% fetal bovine serum (FBS), with the exception of NCI-H792 which was maintained in DMEM supplemented with 10% FBS. The TP53 gene mutation in cell lines was determined by searching the p53 web site (http://p53.free.fr/), the IARC TP53 Database (http://www-p53.iarc.fr/), and the COSMIC database (http://www.sanger.ac.uk/genetics/CGP/cosmic/).

### RNA extraction

Total RNA was extracted from FFPE samples using the RecoverAll Total Nucleic Acid Isolation kit (Ambion, Austin, TX) and total RNA from cell lines was extracted with Trizol reagent (Invitrogen, Carlsbad, CA), in accordance with the manufacturer's instructions.

### Quantitative Real-Time PCR (qRT-PCR)

Expression of mature miRNAs, including miR-21, miR-29b, miR-34a/b/c, miR-155, and let-7a, was evaluated by qRT-PCR analysis as described previously [18]. RNU6B snRNA was used as an endogenous control. Each microRNA assay was performed in triplicate. Expression of microRNAs was reported as delta Ct value (Ct value of RNU6B – Ct value of target microRNA). We defined the groups of tumors or cells with high or low expression based on the median expression value of each microRNA.

Expression of AXL gene was performed by using Taqman gene expression assay (Applied Biosystems) and was reported as delta Ct value. GAPDH gene was used as endogeneous control.

### Transfection of microRNA precursor or inhibitors

miR-34a precursor and inhibitor as well as Cy3-labeled control miR precursor and inhibitor were purchased from Ambion. Transfection of miR-precursor or inhibitor was carried out using siPORT™ *NeoFX*™ Transfection Agent (Ambion) following manufacturer's recommendation at a final oligonucleotide concentration of 30 nM.

### Cell growth analysis

To determine the effect of transfection on cell growth, 5,000–10,000 cells transfected with the indicated microRNA precursor or inhibitor were plated in 96-well plates. Cell growth was determined by CellTiter 96 AQueousOne Solution Cell Proliferation Assay (Promega Corp. Madison, WI) every 24 hours for 5 successive days. Results were presented as the optical density (OD value) absorbance at 490 nm.

### Repetitive transfectin of N592 cell

100,000 NCI-N592 SCLC cells were plated into 24-well plates and transfected with miR-34a precursor or control precursor. Cells were harvested, counted, and transfected again with the respective miRNAs every 72 hours.

### Growth inhibition assays

Cisplatin and etoposide were obtained from Sigma-Aldrich (St. Louis, MI). 10,000 cells were seeded into 96-well flat-bottomed plates, transfected with indicated precursor or inhibitor microRNAs, cultured for 24 hours before the addition of various concentrations of cisplatin and etoposide, and incubated for an additional 72-hour. The cytotoxic effects of cisplatin and etoposide were determined by CellTiter 96 AQueousOne Solution Cell Proliferation Assay. Experiments were performed in triplicate. The percentage of cell viability was determined by dividing the absorbance values of the treated cells by those of the untreated cells, and the concentration of cisplatin or etoposide resulting in 50% growth inhibition (IC_50_) was determined.

### Immunohistochemistry study

Immunohistochemistry study of p53 and bcl-2 in SCLC tumors were performed and reported previously [22].

### Western blot

NCI-N592 and A549 cells transfected with miR-34a precursor or control precursor were incubated for 48 hours. Cells were harvested, lysates were prepared, and Western blot was performed as described previously [34]. Antibodies were obtained from Sigma-Aldrich (actin), Santa Cruz Biotechnology Inc (c-MET), Cell Signaling Technology (MEK1/2), and Dako (bcl-2). The intensities of c-MET and bcl-2 were assessed using GeneTools software (SynGene), normalized by the intensity of actin, and calibrated to the expression level in A549 and NCI-H146 cell, respectively.

### Cell cycle analysis by flow cytometry

48 hours after transfection, cells were collected, washed with cold PBS, fixed with cold 70% ethanol in PBS for at least 24 hours, and labeled with propidium iodide before counting cells. After staining, cells were counted on FACScalibur using the Cellquest Pro software (Becton Dickinson and Company, Frankin Lakes, NJ). Cell cycle fractions were analyzed using the Modfit v3.0 software.

### *In situ* hybridization of microRNAs

*In situ* hybridization of miR-34b was performed as described previously [18]. Nucleolar RNA U6 was used as positive control.

### Statistical analysis

Statistical analysis was performed using SPSS version 17.0 (SPSS, Chicago, IL). Survival was calculated using the Kaplan-Meier method. The comparison of survival between different groups was determined by the log-rank test. The differences in microRNA expression between groups were analyzed by the Wilcoxon Rank-Sum test. The correlation between drug sensitivity and microRNA expression was analyzed by Spearman rank correlation. Comparison of drug sensitivity of cells transfected with various oligonucleotides was carried out by one-way analysis of variances (one-way ANOVA). All p-values were two-sides and p-values less than 0.05 were regarded significant.

## Supporting Information

Figure S1 **Expression of microRNAs in SCLC versus in NSCLC cell lines.** A delta Ct value of −15 indicates that the expression of the microRNA in the cell is too low to be detected.
(TIF)

Figure S2 ***in situ*** **hybridization of microRNAs in a SCLC tumor**. Nucleolar RNA U6 and miR-34b were detected using a biotinyl tyramide-based system with Vector NovaRed as a substrate (brown). Specimens for scramble negative control and miR-34b were co-stained with Mayer's hematoxylin (blue).
(TIF)

Figure S3 **Correlation of expression of p53 and (A) miR-34a, (B)miR-34b, and (C) miR-34c, and (D) correlation of expression of miR-34a and bcl-2.** Expression of p53 and bcl-2 were presented as percentage of stained cells by immunohistochemistry. Correlation coefficient (r) and p-value were determined by Spearman method.
(TIF)

Figure S4 **NCI-H82 cell transfected with (A) Cy3-labeled microRNA inhibitor and (B) Cy3-labeled microRNA precursor.**
(TIF)

Figure S5 **Cell growth assay of NCI-H1299 NSCLC cells transfected with miR-34a or control precursor.** Error bars refer to standard deviations.
(TIF)

Figure S6 **Growth inhibition assay of the GLC4 cells treated with (A) cisplatin and (B) etoposide.** Cells were transfected with the indicated oligonucleotides. Error bars indicate standard deviation of assays in triplicate.
(TIF)

Figure S7 **Protein expression of c-MET and bcl-2 in 5 SCLC and 3 NSCLC cell lines. (B) Correlation between miR-34a and c-MET expression as well as bcl-2 in 5 SCLC cell lines.**
(TIF)

Table S1 **MicroRNAs and clinical covariables.**
(DOC)

Table S2 **Effect of clinical covariables on progression-free survival (PFS).**
(DOC)

Table S3 **Effect of microRNA expression on progression-free survival (PFS, weeks) in SCLC patients stratified by limited disease or extensive disease.**
(DOC)

Table S4 **TP53 status and miR-34a expression in SCLC cell lines.**
(DOC)

Materials S1 **Cancer cell lines.**
(DOC)

## References

[1] Jackman DM, Johnson BE (2005). Small-cell lung cancer.. Lancet.

[2] Govindan R, Page N, Morgensztern D, Read W, Tierney R (2006). Changing epidemiology of small-cell lung cancer in the United States over the last 30 years: analysis of the surveillance, epidemiologic, and end results database.. J Clin Oncol.

[3] Lally BE, Urbanic JJ, Blackstock AW, Miller AA, Perry MC (2007). Small cell lung cancer: have we made any progress over the last 25 years?. Oncologist.

[4] Hurwitz JL, McCoy F, Scullin P, Fennell DA (2009). New advances in the second-line treatment of small cell lung cancer.. Oncologist.

[5] Calin GA, Croce CM (2006). MicroRNA signatures in human cancers.. Nat Rev Cancer.

[6] Iorio MV, Croce CM (2009). MicroRNAs in cancer: small molecules with a huge impact.. J Clin Oncol.

[7] Cho WC (2010). MicroRNAs in cancer - from research to therapy.. Biochim Biophys Acta.

[8] He L, He X, Lim LP, de Stanchina E, Xuan Z (2007). A microRNA component of the p53 tumour suppressor network.. Nature.

[9] Fabbri M, Garzon R, Cimmino A, Liu Z, Zanesi N (2007). MicroRNA-29 family reverts aberrant methylation in lung cancer by targeting DNA methyltransferases 3A and 3B.. Proc Natl Acad Sci U S A.

[10] Cho WC (2010). MicroRNAs: potential biomarkers for cancer diagnosis, prognosis and targets for therapy.. Int J Biochem Cell Biol.

[11] Gallardo E, Navarro A, Vinolas N, Marrades RM, Diaz T (2009). miR-34a as a prognostic marker of relapse in surgically resected non-small-cell lung cancer.. Carcinogenesis.

[12] Johnson SM, Grosshans H, Shingara J, Byrom M, Jarvis R (2005). RAS is regulated by the let-7 microRNA family.. Cell.

[13] Yanaihara N, Caplen N, Bowman E, Seike M, Kumamoto K (2006). Unique microRNA molecular profiles in lung cancer diagnosis and prognosis.. Cancer Cell.

[14] Yu SL, Chen HY, Chang GC, Chen CY, Chen HW (2008). MicroRNA signature predicts survival and relapse in lung cancer.. Cancer Cell.

[15] Markou A, Tsaroucha EG, Kaklamanis L, Fotinou M, Georgoulias V (2008). Prognostic value of mature microRNA-21 and microRNA-205 overexpression in non-small cell lung cancer by quantitative real-time RT-PCR.. Clin Chem.

[16] Olivier M, Eeles R, Hollstein M, Khan MA, Harris CC (2002). The IARC TP53 database: new online mutation analysis and recommendations to users.. Hum Mutat.

[17] Chang TC, Wentzel EA, Kent OA, Ramachandran K, Mullendore M (2007). Transactivation of miR-34a by p53 broadly influences gene expression and promotes apoptosis.. Mol Cell.

[18] Voortman J, Goto A, Mendiboure J, Sohn JJ, Schetter AJ (2010). MicroRNA expression and clinical outcomes in patients treated with adjuvant chemotherapy after complete resection of non-small cell lung carcinoma.. Cancer Res.

[19] Hwang JH, Voortman J, Giovannetti E, Steinberg SM, Leon LG (2010). Identification of microRNA-21 as a biomarker for chemoresistance and clinical outcome following adjuvant therapy in resectable pancreatic cancer.. PLoS One.

[20] Du L, Schageman JJ, Irnov, Girard L, Hammond SM (2010). MicroRNA expression distinguishes SCLC from NSCLC lung tumor cells and suggests a possible pathological relationship between SCLCs and NSCLCs.. J Exp Clin Cancer Res.

[21] Ji Q, Hao X, Zhang M, Tang W, Yang M (2009). MicroRNA miR-34 inhibits human pancreatic cancer tumor-initiating cells.. PLoS One.

[22] Dingemans AM, Witlox MA, Stallaert RA, van der Valk P, Postmus PE (1999). Expression of DNA topoisomerase IIalpha and topoisomerase IIbeta genes predicts survival and response to chemotherapy in patients with small cell lung cancer.. Clin Cancer Res.

[23] Wiggins JF, Ruffino L, Kelnar K, Omotola M, Patrawala L (2010). Development of a lung cancer therapeutic based on the tumor suppressor microRNA-34.. Cancer Res.

[24] Bader AG, Brown D, Winkler M (2010). The promise of microRNA replacement therapy.. Cancer Res.

[25] Fujita Y, Kojima K, Hamada N, Ohhashi R, Akao Y (2008). Effects of miR-34a on cell growth and chemoresistance in prostate cancer PC3 cells.. Biochem Biophys Res Commun.

[26] Ichimura A, Ruike Y, Terasawa K, Shimizu K, Tsujimoto G (2010). MicroRNA-34a inhibits cell proliferation by repressing mitogen-activated protein kinase kinase 1 during megakaryocytic differentiation of K562 cells.. Mol Pharmacol.

[27] Mudduluru G, Ceppi P, Kumarswamy R, Scagliotti GV, Papotti M (2011). Regulation of Axl receptor tyrosine kinase expression by miR-34a and miR-199a/b in solid cancer..

[28] Miko E, Czimmerer Z, Csanky E, Boros G, Buslig J (2009). Differentially expressed microRNAs in small cell lung cancer.. Exp Lung Res.

[29] Ranade AR, Cherba D, Sridhar S, Richardson P, Webb C (2010). MicroRNA 92a-2*: a biomarker predictive for chemoresistance and prognostic for survival in patients with small cell lung cancer.. J Thorac Oncol.

[30] Guo L, Liu Y, Bai Y, Sun Y, Xiao F (2010). Gene expression profiling of drug-resistant small cell lung cancer cells by combining microRNA and cDNA expression analysis.. Eur J Cancer.

[31] Hermeking H (2010). The miR-34 family in cancer and apoptosis.. Cell Death Differ.

[32] Li N, Fu H, Tie Y, Hu Z, Kong W (2009). miR-34a inhibits migration and invasion by downregulation of c-Met expression in human hepatocellular carcinoma cells.. Cancer Lett.

[33] Pang RT, Leung CO, Ye TM, Liu W, Chiu PC (2010). MicroRNA-34a suppresses invasion through downregulation of Notch1 and Jagged1 in cervical carcinoma and choriocarcinoma cells.. Carcinogenesis.

[34] Harada T, Lopez-Chavez A, Xi L, Raffeld M, Wang Y (2011). Characterization of epidermal growth factor receptor mutations in non-small-cell lung cancer patients of African-American ancestry.. Oncogene advance online publication.

