# Supplementary figures and images for: MicroRNA Expression and Clinical Outcome of Small Cell Lung Cancer
Source: PLoS One. 2011 Jun 22;6(6):e21300. doi: 10.1371/journal.pone.0021300 (PMC3120860; doi:10.1371/journal.pone.0021300)

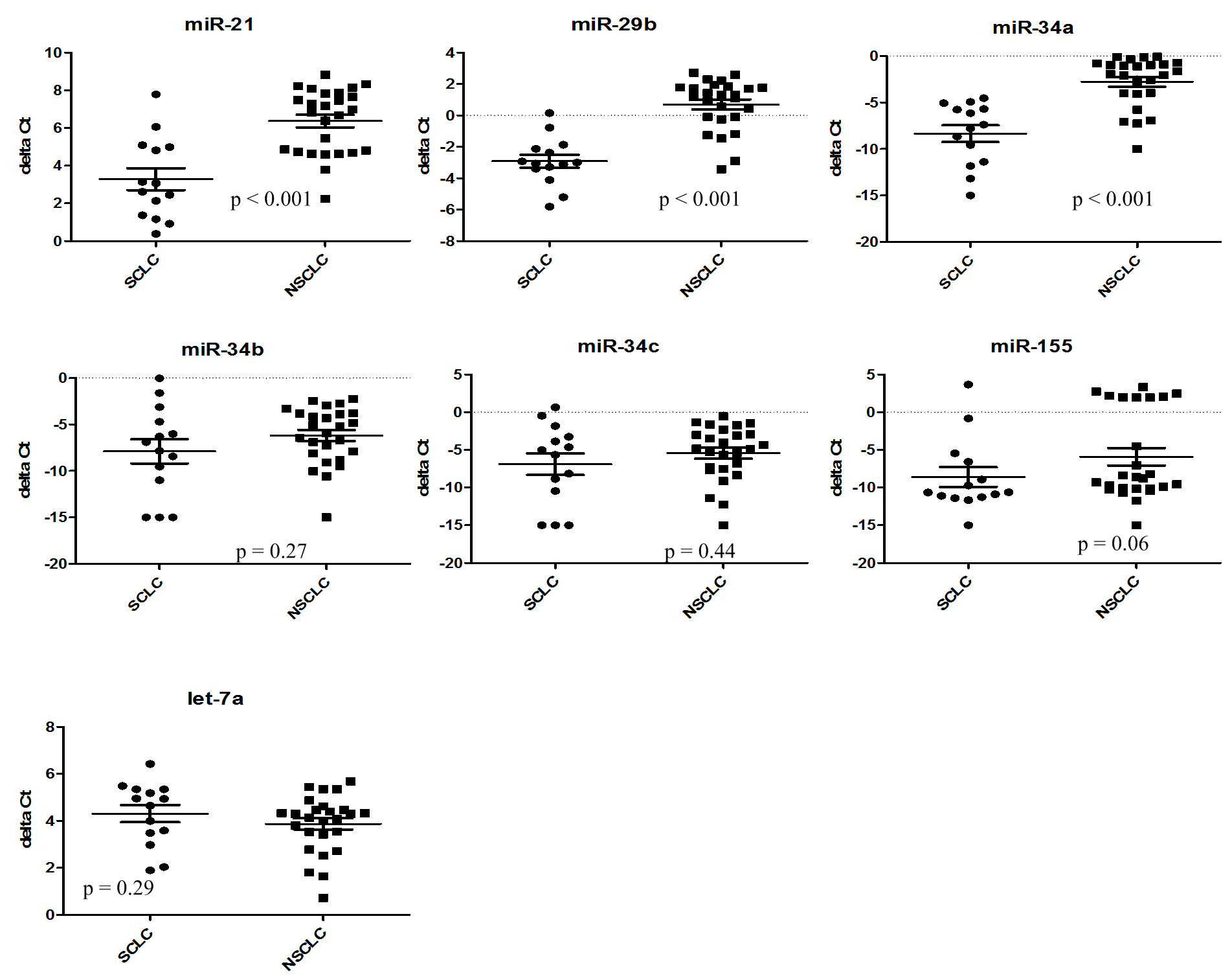

Supplement: Figure S1 — Expression of microRNAs in SCLC versus in NSCLC cell lines. A delta Ct value of −15 indicates that the expression of the microRNA in the cell is too low to be detected. (TIF) [file pone.0021300.s001.tif]

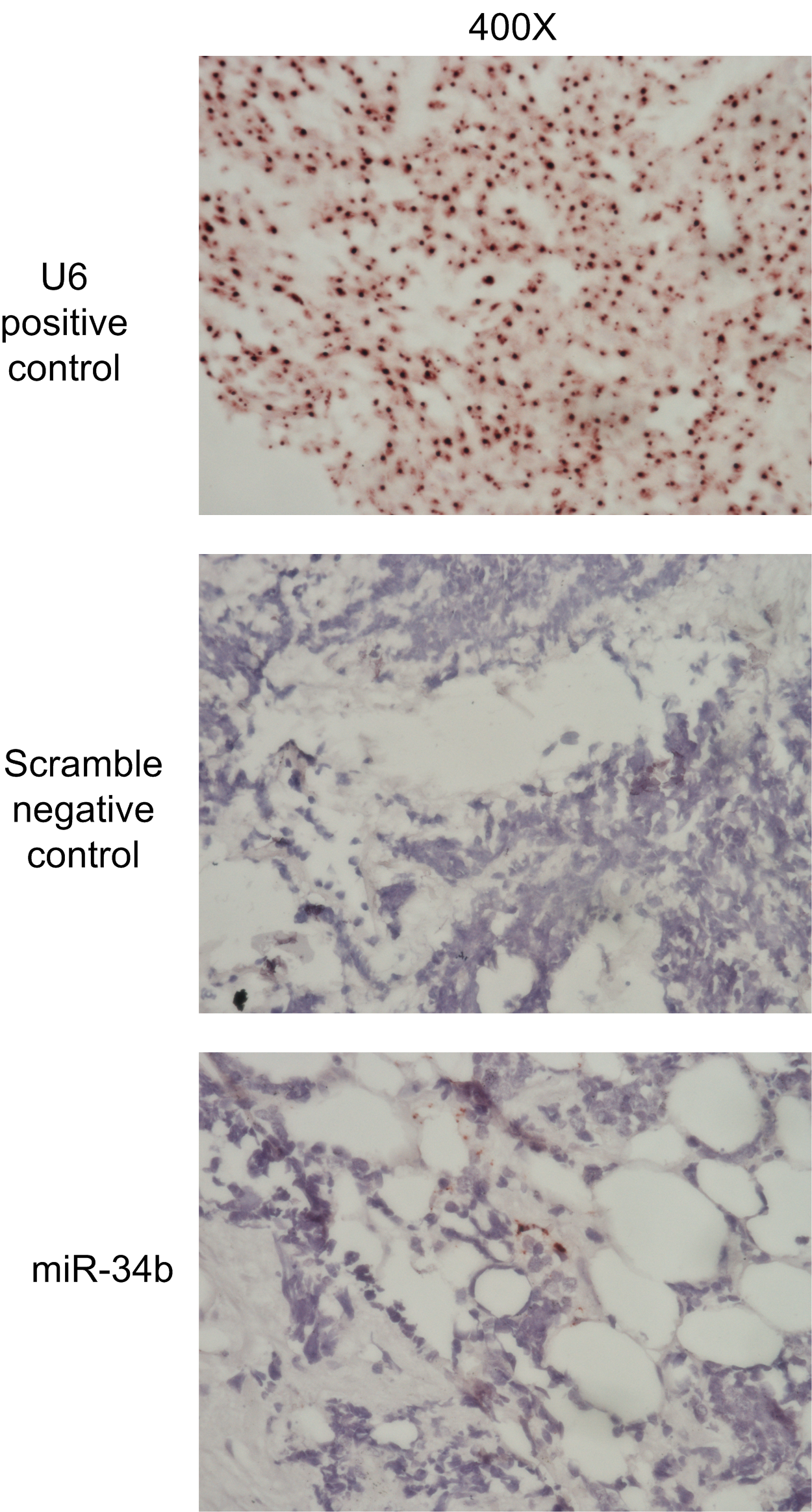

Supplement: Figure S2 — in situ hybridization of microRNAs in a SCLC tumor. Nucleolar RNA U6 and miR-34b were detected using a biotinyl tyramide-based system with Vector NovaRed as a substrate (brown). Specimens for scramble negative control and miR-34b were co-stained with Mayer's hematoxylin (blue). (TIF) [file pone.0021300.s002.tif]

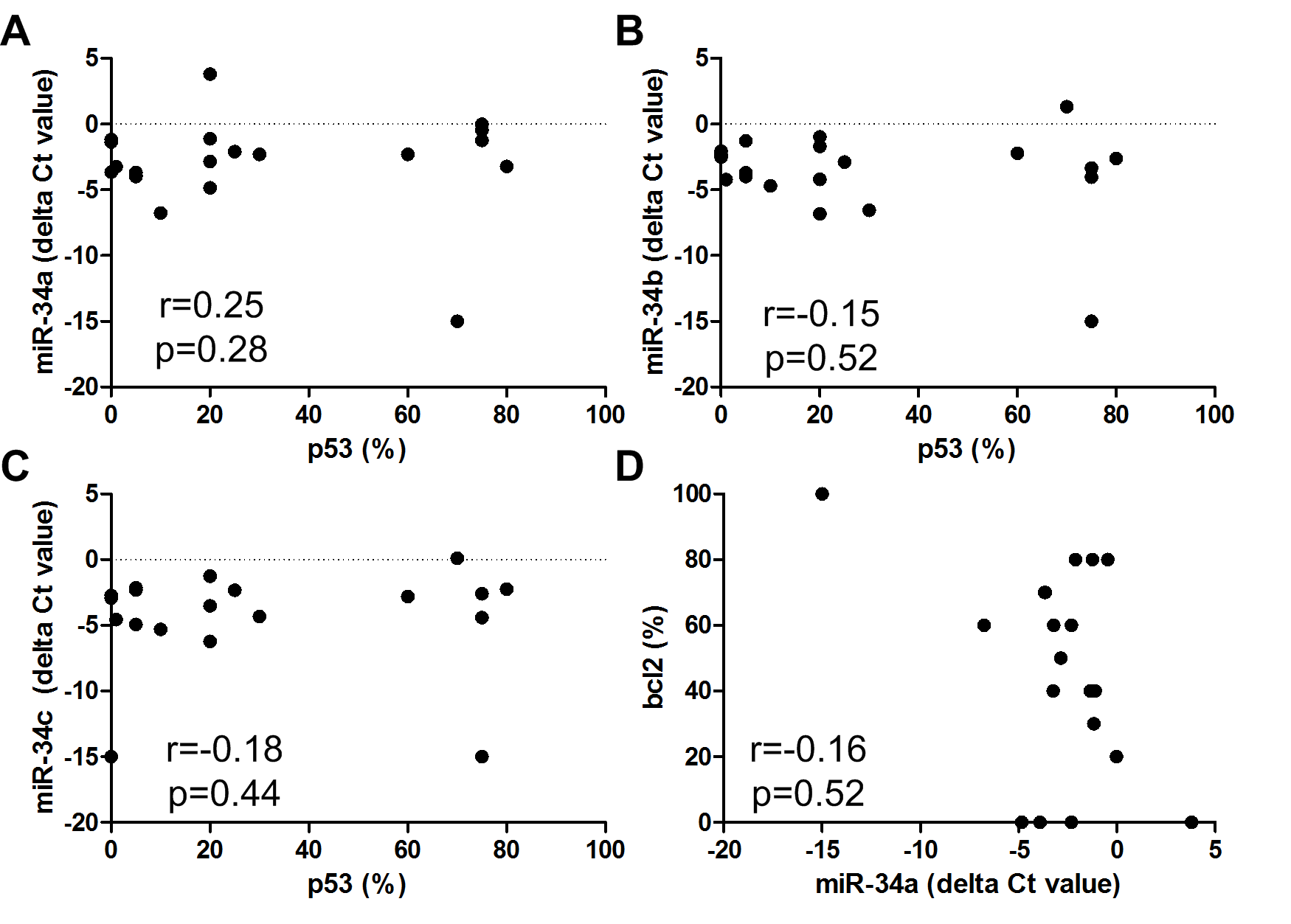

Supplement: Figure S3 — Correlation of expression of p53 and (A) miR-34a, (B)miR-34b, and (C) miR-34c, and (D) correlation of expression of miR-34a and bcl-2. Expression of p53 and bcl-2 were presented as percentage of stained cells by immunohistochemistry. Correlation coefficient (r) and p-value were determined by Spearman method. (TIF) [file pone.0021300.s003.tif]

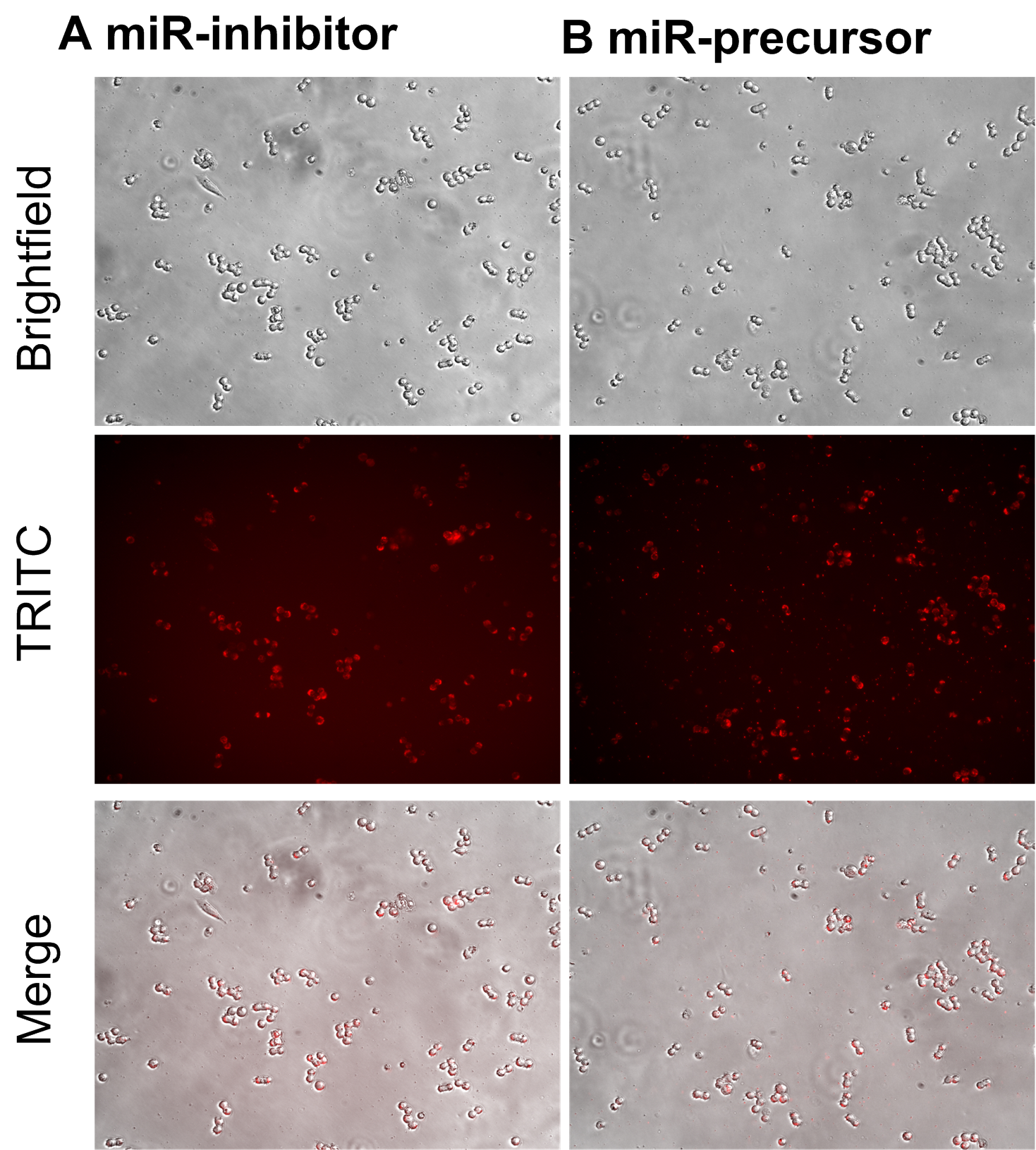

Supplement: Figure S4 — NCI-H82 cell transfected with (A) Cy3-labeled microRNA inhibitor and (B) Cy3-labeled microRNA precursor. (TIF) [file pone.0021300.s004.tif]

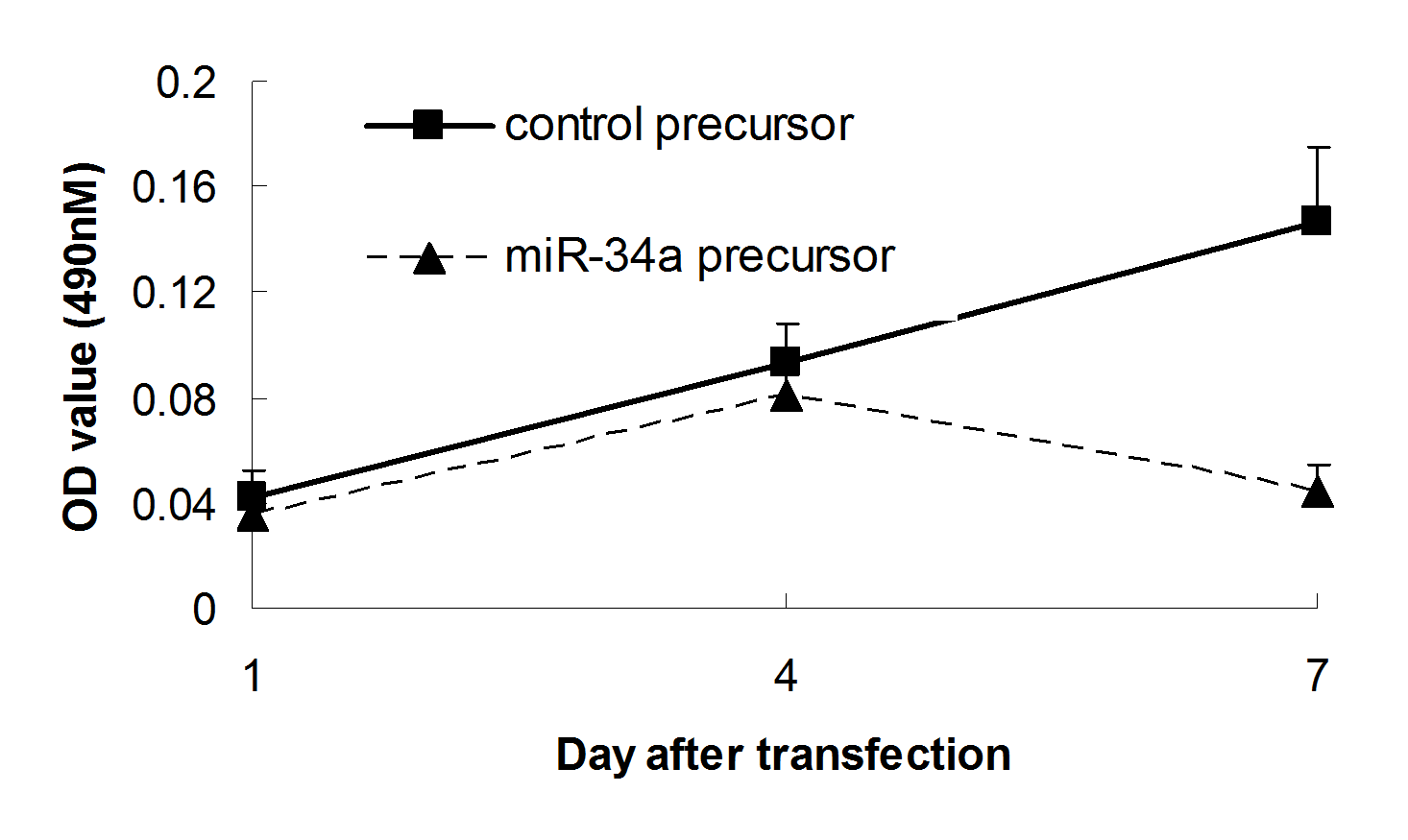

Supplement: Figure S5 — Cell growth assay of NCI-H1299 NSCLC cells transfected with miR-34a or control precursor. Error bars refer to standard deviations. (TIF) [file pone.0021300.s005.tif]

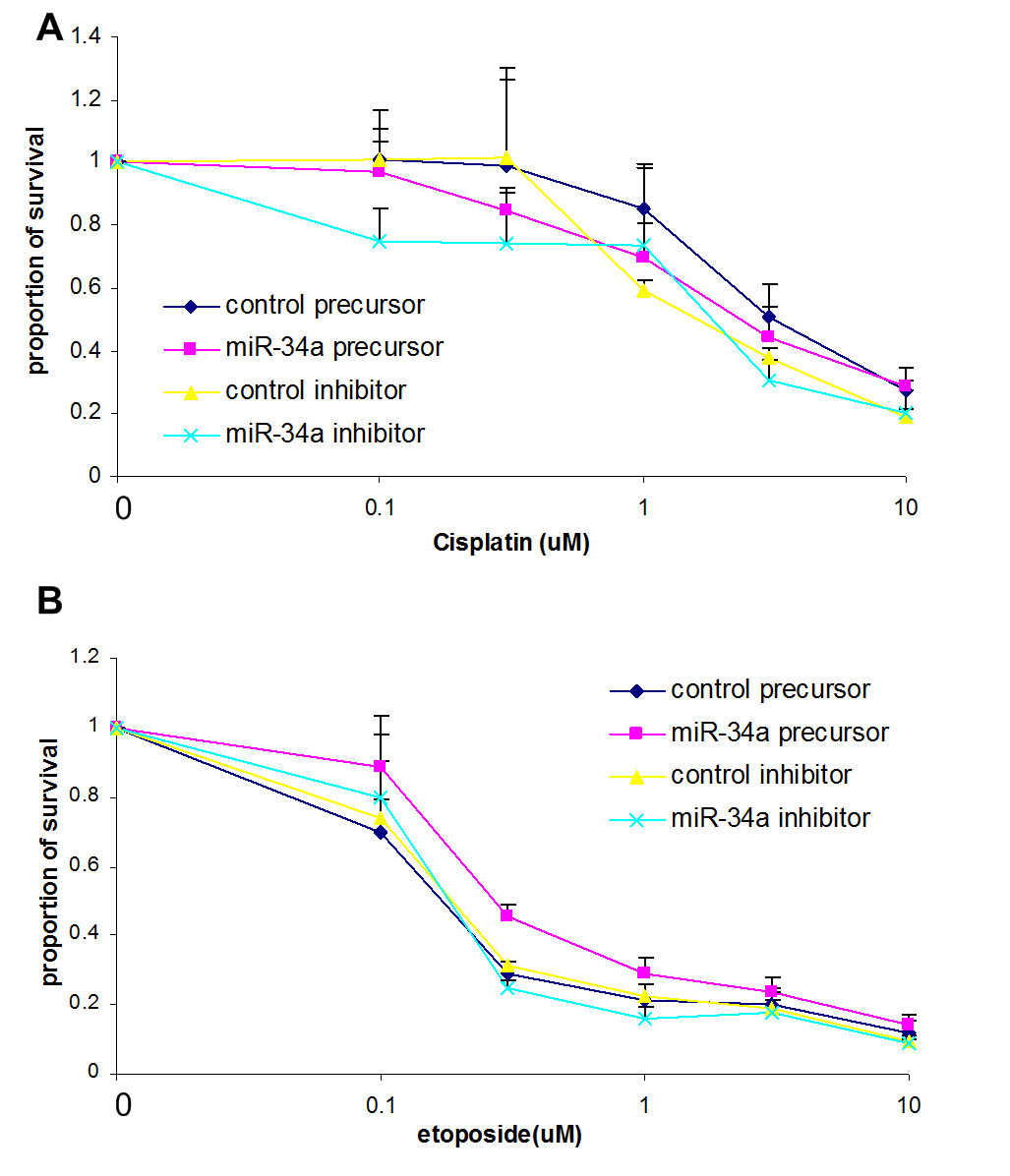

Supplement: Figure S6 — Growth inhibition assay of the GLC4 cells treated with (A) cisplatin and (B) etoposide. Cells were transfected with the indicated oligonucleotides. Error bars indicate standard deviation of assays in triplicate. (TIF) [file pone.0021300.s006.tif]

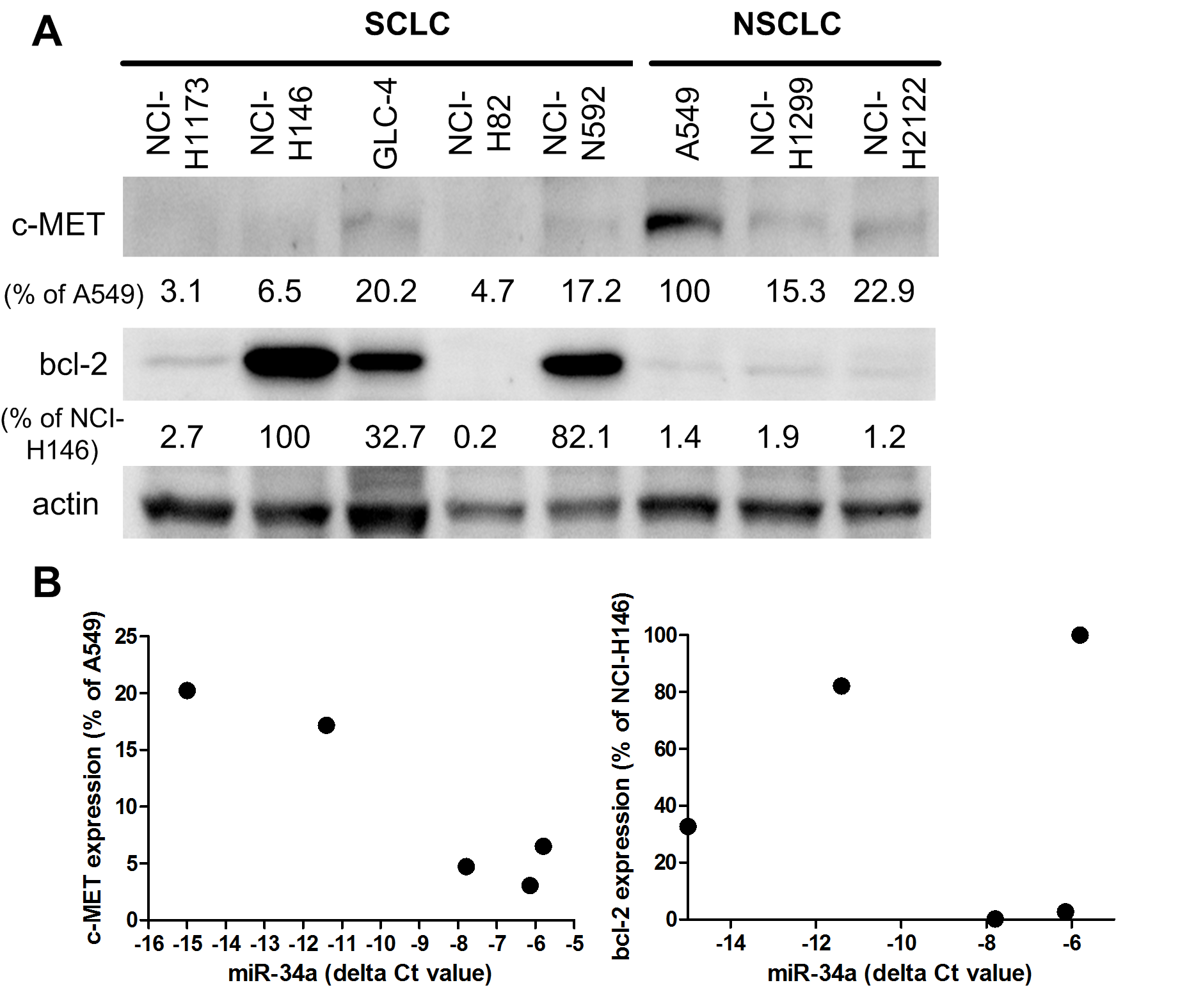

Supplement: Figure S7 — Protein expression of c-MET and bcl-2 in 5 SCLC and 3 NSCLC cell lines. (B) Correlation between miR-34a and c-MET expression as well as bcl-2 in 5 SCLC cell lines. (TIF) [file pone.0021300.s007.tif]
