# Supplementary material for: MicroRNA Expression and Clinical Outcome of Small Cell Lung Cancer
Source: PLoS One. 2011 Jun 22;6(6):e21300. doi: 10.1371/journal.pone.0021300 (PMC3120860; doi:10.1371/journal.pone.0021300)
Supplement: Table S1 — MicroRNAs and clinical covariables. (DOC) [file pone.0021300.s008.doc]

Table S1. MicroRNAs and clinical covariables.

|  | Gender | Age | Disease extent | Treatment regimens | Radiation or not | Complete responder |
| --- | --- | --- | --- | --- | --- | --- |
| miR-21 | .359 | .3 | .439 | .447 | .293 | .398 |
| miR-29b | .641 | .578 | .561 | .447 | .569 | .398 |
| miR-34a | .359 | .106 | .439 | .189 | .431 | .398 |
| miR-34b | .359 | .3 | .561 | .553 | .103 | .602 |
| miR-34c | .359 | .578 | .561 | .447 | .023 | .602 |
| miR-155 | .359 | .3 | .189 | .447 | .569 | .602 |
| let-7a | .011 | .024 | .439 | .05 | .431 | .602 |
| Expression of each microRNA were divided into high expression group and low expression group according to the median. Clinical variables were categorized as those in table 1 The numbers represent p-value determined by chi-square test | | | | | | |
