# Supplementary material for: MicroRNA Expression and Clinical Outcome of Small Cell Lung Cancer
Source: PLoS One. 2011 Jun 22;6(6):e21300. doi: 10.1371/journal.pone.0021300 (PMC3120860; doi:10.1371/journal.pone.0021300)
Supplement: Table S2 — Effect of clinical covariables on progression-free survival (PFS). (DOC) [file pone.0021300.s009.doc]

Table S2. Effect of clinical covariables on progression-free survival (PFS).

|  |  | PFS (weeks) | p-value |
| --- | --- | --- | --- |
| gender | male | 36.1 | 0.213 |
|  | female | 19.4 |  |
| age | ≦ median | 33.9 | 0.767 |
|  | > median | 36.1 |  |
| extent | LD | 42 | 0.015 |
|  | ED | 26.1 |  |
| treatment | CEE | 37.4 | 0.165 |
|  | CEV/PE | 32.7 |  |
| radiation | Yes | 36.7 | 0.041 |
|  | No | 12.5 |  |
| response | CR | 54.7 | <0.001 |
|  | non-CR | 30.1 |  |
| Abbreviation: CR, complete responder; LD, limited disease; ED, entensive disease; CEE, cyclophosphamide-epirubicin-etoposide; CEV/PE, cyclophosphamide -epirubicine-vincristine alternated with carboplatin-etoposide. | | | |
