# Supplementary material for: MicroRNA Expression and Clinical Outcome of Small Cell Lung Cancer
Source: PLoS One. 2011 Jun 22;6(6):e21300. doi: 10.1371/journal.pone.0021300 (PMC3120860; doi:10.1371/journal.pone.0021300)
Supplement: Table S3 — Effect of microRNA expression on progression-free survival (PFS, weeks) in SCLC patients stratified by limited disease or extensive disease. (DOC) [file pone.0021300.s010.doc]

Table S3. Effect of microRNA expression on progression-free survival (PFS, weeks) in SCLC patients stratified by limited disease or extensive disease.

|  |  | Limited disease | | | Extensive disease | | |
| --- | --- | --- | --- | --- | --- | --- | --- |
| microRNA |  | no. | PFS | p-value | no. | PFS | p-value |
| miR-21 | high | 10 | 24.0 | 0.68 | 6 | 19.6 | 0.35 |
|  | low | 8 | 34.9 |  | 7 | 26.1 |  |
| miR-29b | high | 9 | 31.9 | 0.48 | 7 | 26.1 | 0.52 |
|  | low | 9 | 26.2 |  | 6 | 24.0 |  |
| miR-34a | high | 10 | 38.4 | 0.53 | 6 | 19.4 | 0.36 |
|  | low | 8 | 42.0 |  | 7 | 31.4 |  |
| miR-34b | high | 9 | 43.3 | 0.24 | 7 | 26.1 | 0.90 |
|  | low | 9 | 38.4 |  | 6 | 19.4 |  |
| miR-34c | high | 9 | 45.9 | 0.95 | 7 | 31.4 | 0.17 |
|  | low | 9 | 34.9 |  | 6 | 18.9 |  |
| miR-155 | high | 11 | 42.0 | 0.41 | 5 | 19.6 | 0.69 |
|  | low | 7 | 43.3 |  | 8 | 26.1 |  |
| let-7a | high | 10 | 38.4 | 0.65 | 6 | 19.4 | 0.22 |
|  | low | 8 | 42.0 |  | 7 | 35.7 |  |
