# Supplementary material for: MicroRNA Expression and Clinical Outcome of Small Cell Lung Cancer
Source: PLoS One. 2011 Jun 22;6(6):e21300. doi: 10.1371/journal.pone.0021300 (PMC3120860; doi:10.1371/journal.pone.0021300)
Supplement: Table S4 — TP53 status and miR-34a expression in SCLC cell lines. (DOC) [file pone.0021300.s011.doc]

**Table S4. TP53 status and** miR-34a expression in SCLC cell lines

| Cells | TP53 gene mutation | delta Ct | High/low expression |
| --- | --- | --- | --- |
| NCI-H69 | p.E171(stop) | -4.53 | High |
| NCI-H82 | p.T125T | -7.79 | Low |
| NCI-H187 | p.S241C | -11.83 | Low |
| NCI-H526 | c.97-1G>C(intronic) | -5.06 | High |
| NCI-N592 |  | -11.37 | Low |
| NCI-H620 |  | -7.38 | High |
| NCI-H678 |  | -8.66 | Low |
| NCI-H792 |  | -5.69 | High |
| NCI-H1173 | c.994-1G>C(intronic) | -6.14 | High |
| NCI-H128 | WT | -4.92 | High |
| NCI-H146 | p.P318(frame shift) | -5.76 | High |
| GLC-4 | p.K132E | <-15 | Low |
| GLC4-CDDP |  | -11.35 | Low |
| AC-3 |  | -13.17 | Low |
| NCI-H69 and NCI-N592 were established from the same patient | | | |
