# Supplementary material for: MicroRNA Expression and Clinical Outcome of Small Cell Lung Cancer
Source: PLoS One. 2011 Jun 22;6(6):e21300. doi: 10.1371/journal.pone.0021300 (PMC3120860; doi:10.1371/journal.pone.0021300)
Supplement: Materials S1 — Cancer cell lines. (DOC) [file pone.0021300.s012.doc]

**Supporting Materials S1**

**Cancer cell lines.** The SCLC cells were GLC4 and GLC4-CDDP (kindly provided by Dr. S. de Jong, Groningen, Netherlands), NCI-H69, NCI-H82, NCI-H128, NCI-H146, NCI-H187, NCI-H526, NCI-N592, NCI-H620, NCI-H678, NCI-H792, NCI-H1173, and AC-3. The NSCLC cell lines were A549, CORL23, NCI-H125, NCI-H157, NCI-H322, NCI-H358, NCI-H441, NCI-H460, NCI-H820, NCI-H838, NCI-H854, NCI-H1264, NCI-H1299, NCI-H1355, NCI-H1373, NCI-H1466, NCI-H1717, NCI-H1725, NCI-H1944, NCI-H2077, NCI-H2087, NCI-H2122, NCI-H2250, NCI-H2347, NCI-H3123, and NCI-H3255.
